# Remodeling Effects of the Combination of GGT Scaffolds, Percutaneous Electrical Stimulation, and Acupuncture on Large Bone Defects in Rats

**DOI:** 10.3389/fbioe.2022.832808

**Published:** 2022-02-28

**Authors:** Chun-Hsu Yao, Bo-Yin Yang, Yi-Chen Ethan Li

**Affiliations:** ^1^ School of Chinese Medicine, College of Chinese Medicine, Graduate Institute of Chinese Medicine, China Medical University, Taichung, Taiwan; ^2^ Department of Biomedical Imaging and Radiological Science, China Medical University, Taichung, Taiwan; ^3^ Biomaterials Translational Research Center, China Medical University Hospital, Taichung, Taiwan; ^4^ Department of Biomedical Informatics, Asia University, Taichung, Taiwan; ^5^ Department of Chemical Engineering, Feng Chia University, Taichung, Taiwan

**Keywords:** tricalcium phosphate (TCP)-based gelatin scaffold, bone remodeling, electrical stimulation, acupuncture, osteogenesis

## Abstract

The regeneration defect of bone is a long-term physiological process after bone injuries. To accelerate the bone remodeling process, the combination of chemical and physical stimulations provides an efficient strategy to allow maturation and to functionalize osteoclasts and osteoblasts. This study aims to investigate the dual effects of a tricalcium phosphate (TCP)-based gelatin scaffold (GGT) in combination with electroacupuncture stimulation on the activation of osteoclasts and osteoblasts, as well as new bone regrowth *in vitro and in vivo*. We demonstrated that electrical stimulation changes the pH of a culture medium and activates osteoblasts and osteoclasts in an *in vitro* co-culture system. Furthermore, we showed that electroacupuncture stimulation can enhance osteogenesis and new bone regrowth *in vivo* and can upregulate the mechanism among parathyroid hormone intact (PTH-i), calcium, osteoclasts, and osteoblasts in the bone-defected rats. Those results showed the potential interest to combine the electroacupuncture technique with GGT scaffolds to improve bone remodeling after injury.

## Introduction

Large bone injuries can occur in degenerative/cancer diseases, skeletal trauma, or elderly people (Dang et al., 2018). To promote bone reconstruction, several strategies are well developed. For example, autologous and allogeneic bone transplantations are considered as the gold standards for bone reconstruction (Hou et al., 2014; Verrier et al., 2016). However, bone transplantation suffers from the limitations of available tissue, potential immune-related complications, and costly sample preparation (Dimitriou et al., 2011; Vidal et al., 2020). Therefore, artificial scaffolds such as porous sponge or extracellular matrix (ECM)-based substrates have been investigated as an alternative to promote the efficiency of regeneration. For example, Wang et al. have combined polycaprolactone (PCL) and mesoporous silicate nanoparticles (MSNs) to develop a nanofibrous scaffold *via* an electrospinning technology. This PCL-based nanofibrous scaffold transplanted into rats significantly enhances bone remodeling within 4 weeks (Wang et al., 2018). The ECM contains tissue-specific proteins with mechanical and structural properties secreted from cells. The ECM-based scaffold allows stem or somatic cells from original tissues to migrate into the scaffold and then repair the injured tissues (Edgar et al., 2016). Mattioli-Belmonte et al. have developed a decellularized and demineralized bovine bone scaffold retaining both the chemical matrix cues and the topological features of native tissues (Mattioli-Belmonte et al., 2019). The cell-free decellularized tissue provides human umbilical cord-derived mesenchymal stem cells (hUC-MSCs) with a new supporting matrix to migrate and spread into the bone defect area before undergoing osteogenic differentiation.

Cytokines such as growth factors and chemical compounds are also used as a strategy to induce the healing of injured bone tissues. Bone morphogenetic proteins (BMPs) such as BMP2, BMP7, and BMP9 have been demonstrated to promote osteogenetic or condrogenetic differentiation of MSCs *in vitro* and *in vivo* (Beederman et al., 2013). The transforming growth factor-β (TGF-β) family has been reported to be involved in osteogenesis, skeletal development, and complex bone remodeling through crosstalk with different signaling pathways (Wu et al., 2016; Xu et al., 2018). Bioactive ceramics such as hydroxyapatite (HA) and tricalcium phosphate (TCP) are chemical compounds used as bone substitutes to induce osteogenesis (Lee et al., 2013). These studies have shown that the chemical induction of growth factors or bioactive chemical compounds is useful in bone tissue remodeling.

Recently, electromagnetic stimulation has been used as an adjunctive treatment for bone regeneration (Ebrahim et al., 2014). A previous study has reviewed and showed that electrical stimulation (ES) can regulate the proliferation, migration, and osteogenesis of cells in bone tissue engineering; moreover, the response of cells to ES was also involved in many cellular signaling pathways (Leppik et al., 2020). Through electromagnetic stimulation, some signaling pathways such as the calcium–calmodulin pathway may be activated, leading to the upregulation of cytokines involved in bone remodeling such as TGF-β or BMP (Aleem et al., 2016). The electromagnetic stimulation is conducted through percutaneous ES with stainless steel needles connected to the electrodes. Interestingly, in traditional Chinese medical therapy, acupuncture is also performed using needles to percutaneously reach the acupoints. Although the molecular mechanism involved in acupuncture is still not clear, previous studies have reported that the use of acupuncture as an adjunctive therapy could reduce neuropathic pain and promote ulna bone healing (Ju et al., 2013; Naddaf et al., 2014). These reports indicate acupuncture as an adjunctive therapy that may be useful for bone engineering. Our previous study has combined electromagnetic stimulation (i.e., physical treatment) and the TCP-based gelatin scaffold (GGT) (i.e., chemical compound) to accelerate the reconstruction effect of large bone defects (Yang et al., 2013). However, our previous study used only one ES condition (2 Hz and 2 mA) to treat the rats with bone defects and do not investigate the mechanism of bone healing when performing both a physical treatment and a chemical compound on rats. In this study, we evaluated the combination of the TCP-based gelatin scaffold (GGT) with a dual physical treatment, ES and acupuncture (called electroacupuncture), on bone regeneration. Different from our previous study (Yang et al., 2013), we systematically investigated the effect of the frequency and the current ampere of ES on an *in vitro* osteogenic model and rats and further discussed the relative mechanism among the GGT, ES, and acupuncture in bone regeneration. We assume that the combination of the GGT and electroacupuncture may enhance the expression of molecules and cell activities involved in bone remodeling *in vitro* or *in vivo.*

## Materials and Methods

### Implant Material Preparation

Type A gelatin (50,000–100,000 Da) was purchased from Sigma-Aldrich, United States An 18% gelatin solution was dissolved in distilled water at 70^∘^C. When the gelatin solution was cooled to 50^°^C, a 20% genipin solution (Challenge Bioproducts Co., Taichung, Taiwan) was added to the gelatin solution at a constant temperature for a crosslink reaction. Then, ceramic tricalcium phosphate Ca_3_(PO_4_)_2_ particles of around 200–300 μm (Merck, Germany) were added into the gelatin–genipin mixture. To mimic an inorganic/organic ratio in the natural bone, the TCP and gelatin in the composite were at a weight ratio of 3:1 (Yao et al., 2005). The GGT composites for the animal experiment in this study were prepared with a shape width of 8 mm, and their thickness was 1.5 mm. All samples were stored at −80∘C for 24 h and then dried in a freeze dryer for another 24 h.

### *In Vitro* Preosteoblast (OB)/Preosteoclast (OC) Co-culture System

An OB cell line (MC3T3-E1) and an OC cell line (Raw 264.7) were used in this study. The *in vitro* OB/OC co-culture system was modified by a previous study (Satue et al., 2013) and our previous OB/OC co-culture protocol (Chen et al., 2013). The OB and OC cells were individually cultured in the medium containing the receptor activator of the NF-κB ligand (RANKAL) and the monocyte chemotactic and stimulating factor (M-CSF) for 6 days of incubation. During the individual culture period, the culture medium was refreshed every 2 days to induce the maturation of cells. Afterward, the two mature cells were harvested in the following co-culture experiments. The two cell lines were directly co-cultured and came in contact with each other in a culture dish at 3 × 10^5^ cells individually. Then, the co-culture cells were treated by the medium with RANKAL and M-CSF for 6 days. Then, two dependent OB/OC co-cultured dishes were connected with a salt bridge that consisted of 1.2% agarose and *a*-MEM. Furthermore, *via* the connection of the cathode and anode, the co-cultured cells were stimulated at various sixteen frequency/ampere conditions (1 Hz/1 mA, 1 Hz/4 mA, 1 Hz/8 mA, 2 Hz/1 mA, 2 Hz/4 mA, 2 Hz/8 mA, 20 Hz/1 mA, 20 Hz/4 mA, 20 Hz/8 mA, 200 Hz/1 mA, 200 Hz/4 mA, and 200 Hz/8 mA). During the stimulation period, the co-cultured cells were stimulated for 15 min each time and three times/6 days (Days 0, 3, and 6). Then, the pH value of the culture medium from the co-cultured dish, alkaline phosphatase (ALP), and tartrate-resistant acid phosphatase (TRAP) was analyzed after treatment of ES.

### Surgical Procedure

Twenty adult male Sprague–Dawley rats weighing 280–300 g were used for cranial implantation. The care and experimental protocol of animals followed the national animal care guidelines and was approved by the Institutional Animal Care and Use Committee (IACUC) of China Medical University. Isoflurane (Abbott, Taiwan) was used as an anesthetic agent for all animals. Prior to begin the treatment, the head of each rat was shaved, the head skin was incised in a T-shape, and the parietal periosteum was further removed. Then, a slow-speed dental handpiece with a drilling burr was used to create a full-thickness defect of the parietal bone. An 8-mm circular injury was created on the parietal bone; the dura and the superior sagittal sinus were not violated. Then, the GGT composite scaffolds were used to fill the injuries in the rats. Furthermore, the periosteum and skin were closed by using 5–0 vicryl and 3–0 black silk sutures, respectively (Dong et al., 2008).

### Percutaneous Electrical Stimulation Procedure

**FIGURE 1:**
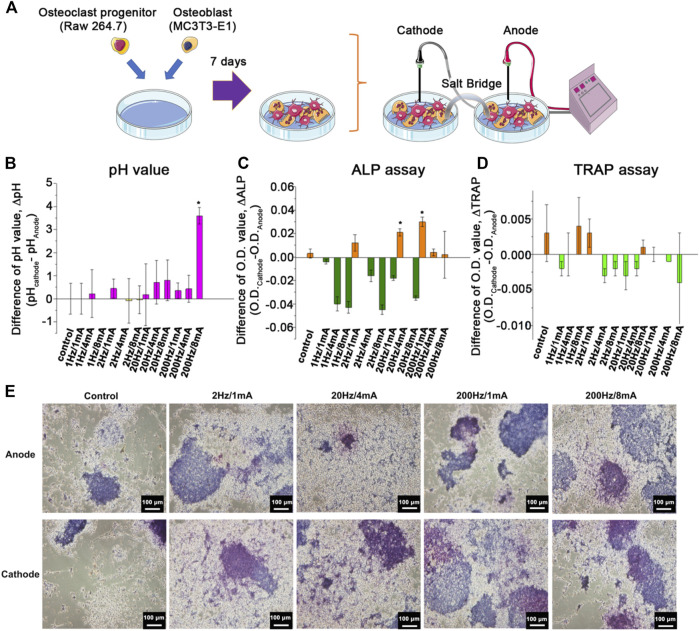
**(A)** Schematic figure of an *in vitro* OB/OC co-culture system treated with ES. The difference of **(B)** the pH value (ΔpH) of the medium, **(C)** ALP expression (ΔALP), and **(D)** TRAP expression (ΔTRAP) of the co-cultured cells at the cathode and anode site after treating ES with 1 Hz/1 mA, 1 Hz/4 mA, 1 Hz/8 mA, 2 Hz/1 mA, 2 Hz/4 mA, 2 Hz/8 mA, 20 Hz/1 mA, 20 Hz/4 mA, 20 Hz/8 mA, 200 Hz/1 mA, 200 Hz/4 mA, and 200 Hz/8 mA. **(E)** The histological analysis of the ALP expression of the co-cultured cells at the cathode and anode site without ES and after treating ES with 2 Hz/1 mA, 2 Hz/4 mA, 20 Hz/4 mA, 200 Hz/1 mA, and 200 Hz/8 mA. The control group indicated the cells treated with ES treatment on the culture dish without the GGT scaffold. (*n* = 6, *p < 0.05.)

Percutaneous electrical stimulation was performed based on our previous study (Yang et al., 2013). Two acupoints Baihui (GV20) and Fengfu (GV16) were selected for ES treatment. After the operation, the rats were divided into five groups, with four rats per group. All groups underwent 4, 8, and 12 weeks of percutaneous electrical stimulation [15 min each time and three times/6 days (Days 0, 3, and 6)] using stainless steel needles (0.27 mm OD, 13 mm length, Ching Ming, Taiwan) with an insertion depth of 2 mm and a stimulator (Trio 300; Ito, Tokyo, Japan). The anode was connected to a point on the back of the neck; the cathode was connected to a front point on the head, as illustrated in Figure 1. During each stimulation, the rats were under inhalation anesthesia. GGT groups without ES, as control groups, only received inhalation anesthesia without percutaneous electrical stimulation. All the rats were examined afterward at 4, 8, and 12 weeks.

### Harvesting, Radio Morphometry, and Histomorphometry of Tissues

In this study, we selected the four groups with the increased DALP expression (i.e., 2Hz/1mA, 20Hz/4mA, 200 Hz/1 mA, and 200 Hz/8 mA) from the *in vitro* co-culture system for the following animal experiments. The rats with the implanted GGT scaffold without the ES treatment were used as the control group. Furthermore, the bone defect regeneration was evaluated radiographically and histologically. Using a micro-CT scanner (SkyScan-1076, Aartselaar, Belgium) and with inhalation anesthesia, each group of animals was examined 4, 8, and 12 weeks after individual percutaneous electrical stimulation. The contrast of gray levels between the new bone tissues and the implanted GGT scaffold was enhanced, and ImageJ (National Institutes of Health, United States) was used to evaluate the volume of the newly formed bone *via* counting the number of voxels. To evaluate the growth trend, 3D images of the new bone were reconstructed through Amira (Visage Imaging GmbH, Berlin, Germany).

To analyze the new bone growth, the anesthetized animals were sacrificed by using carbon dioxide. The removed craniectomy sites with 2–3 mm of the contiguous bone from each skull were immersed into phosphate-buffered 10% formalin for 24 h of fixation. Subsequently, the fixed specimens were placed into the 10% formic acid solution to decalcify all of the calvarial specimens for 2 weeks. After treatment for 2 weeks, the calvarial specimens were immersed in sodium sulfate overnight, dehydrated in a graded series of ethanol, and further embedded in a tissue-freezing medium (OCT). Axial sections of the decalcified bone and implants (10 μm thickness each) were prepared and stained with hematoxylin and eosin (H and E). To observe the relationship between the electrodes and osteoblasts or osteoclasts, longitudinal sections of other specimens (10 μm thickness each) were arranged and stained with either the alkaline phosphatase (ALP) stain or tartrate-resistant acid phosphatase (TRAP). Photomicrographs of these sections were obtained by light microscopy. Furthermore, to quantify the results of the ALP expression, p-Nitrophenyl phosphate (pNPP) from the ALP assay kit (BioVision, Taiwan) and acid phosphatase activity colorimetric assay kit (BioVision, Taiwan) was used to react with ALP and TRAP. After the reaction, the degree of ALP and TRAP activities was further measured at 405 nm by using a plate reader. Moreover, the ΔALP or ΔTRAP expression was calculated by the difference from the O.D. values between the cathode and anode.

### Mathematical Model Simulation

The normal bone regeneration model is modified from previous studies (Komarova et al., 2003; Ayati et al., 2010). The bone cell population in a bone regeneration system could be described by an ordinary differential equation (ODE). The following ODE contains the bone resorption of osteoclasts and the bone formation of osteoblasts, which are expressed in the density of osteoclasts *C(t)* and osteoblasts *B(t)* at the time (t) as below:

$$\frac{d}{dt}\;C\left(t\right)=\alpha_{1}C{\left(t\right)}^{g_{11}}B{\left(t\right)}^{g_{21}}-\beta_{1}C\left(t\right),$$ (1)

$$\frac{d}{dt}\;B\left(t\right)=\alpha_{2}C{\left(t\right)}^{g_{11}}B{\left(t\right)}^{g_{22}}-\beta_{2}B\left(t\right).$$ (2)

In Eq. 1 and 2, the proliferation terms of osteoclasts and osteoblasts are expressed in the power nonlinear terms. *a* is the activity of cell production, and *ß* is the activity of cell removal. g_11_ and g_21_ are the positive and negative feedbacks on the generation of osteoclasts in autocrine and paracrine signaling, respectively. Similarly, g_22_ and g_12_ are both the positive feedbacks on the generation of osteoblasts in autocrine and paracrine signaling, respectively. Therefore, the increase rate of osteoclast and osteoblast numbers above the steady state could affect the rate of bone resorption and regeneration. Therefore, the related rate of bone resorption and regeneration could be assumed as the following equation while over steady-state levels.

$$\frac{d}{dt}\;Z\left(t\right)=\;-k_{1}\max\left[0,\;C\left(t\right)-\bar{C}\right]+k_{2}\max\left[0,\;B\left(t\right)-\bar{B}\right].$$ (3)

Z is the total bone mass; the initiation conditions of Eq. 3 are Z (0) = Z_0_, and the value of k_1_ and k_2_ indicates the normalized activity of bone resorption and regeneration.

### Statistical Analysis

All numerical data were presented as mean ± one standard deviation. Significant differences among the samples were evaluated using one-way ANOVA followed by Tukey’s test to compare data. Probabilities of *p* < 0.05 were considered statistically significant.

## Results

### The Effect of Electrical Stimulation on Osteoblast and Osteoclast Co-Cultured System

To optimize the ES condition, the *in vitro* OB/OC co-culture system was used to investigate the effects of various frequencies and currents of ES on cell behaviors (Figure 1A). The previous study reported that the pH of the culture medium could be impacted by ES and that pH variation may affect cell behaviors (Li et al., 2015). Figure 1B shows a difference of the pH value (ΔpH) measured in the culture medium. After ES treatment, the positive value of ΔpH indicates alkalization of the culture medium. Increased pH of the culture medium was observed when the OB/OC co-culture dish received the frequencies and currents at 1 Hz/4 mA, 2 Hz/1 mA, 20 Hz/4 mA, 20 Hz/8 mA, 200 Hz/1 mA, 200 Hz/4 mA, and 200 Hz/8 mA. The 200 Hz/8 mA group showed a significantly higher ΔpH than other groups.

ALP is an early marker of osteoblast differentiation. In general, a high ALP expression indicates a high degree of mineralization of the bone. Next, the ALP expression of osteoblasts in the OB/OC co-culture dish was analyzed following the ES treatment after 3 days of incubation. Similar to the pH assay, the ALP expression was described as the difference of the ALP expression (ΔALP) (i.e., the O.D. _cathode_ - O.D. _anode_). As shown in Figure 1C, an increased ΔALP expression was observed in the ES treatment group at 2 Hz/1 mA, 20 Hz/4 mA, 200 Hz/1 mA, 200 Hz/4 mA, and 200 Hz/8 mA.

In addition to the osteoblast ALP expression, the TRAP expression was also evaluated as ES might impact osteoclast behaviors (Nakamura et al., 2016). As shown in Figure 1D, ES treatment can induce the expression of TRAP with the following parameters: 1 Hz/8 mA, 2 Hz/1 mA, 2 Hz/8 mA, and 20 Hz/1 mA. Moreover, Figure 1E shows a consistent result with the quantitative results of the ALP expression (Figure 1C) in which ES treatment induces a higher ALP expression. Based on those results, the following parameters were selected to confirm the ES effect *in vivo:* 2 Hz/1 mA, 20 Hz/4 mA, 200 Hz/1 mA, and 200 Hz/8 mA.

### Combination of the Effects of GGT, ES, and Acupuncture on Rats With Bone Defects

**FIGURE 2:**
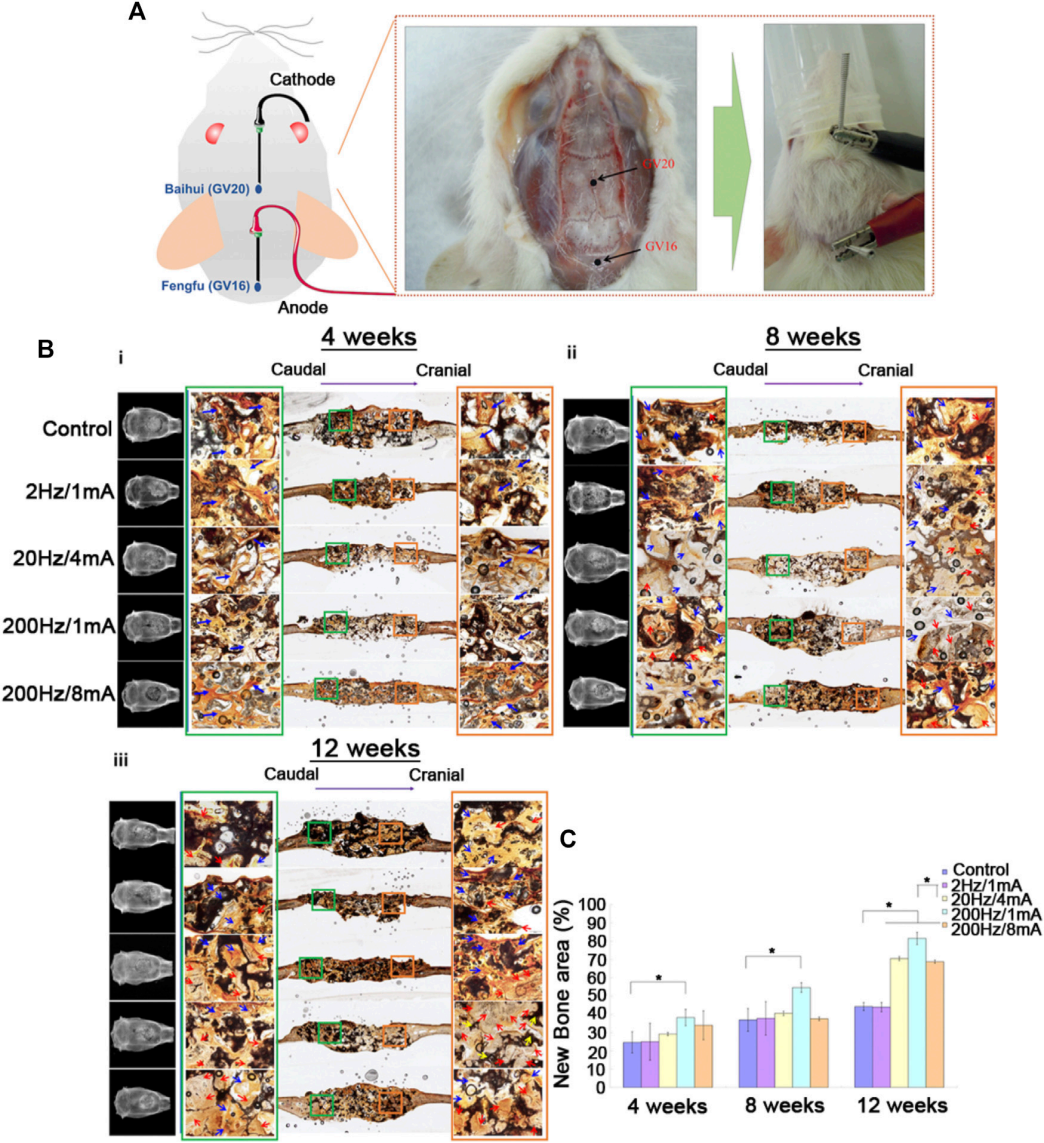
**(A)** Photographic images of the bone-defected rats with the implanted GGT scaffold undergoing electroacupuncture stimulation on Baihui (GV20) and Fengfu (GV16) acupoints. [**(B)** (i)-(iii)] The micro-CT and histological images of the new bone regenerative behavior of rats were treated with electroacupuncture stimulation for 4, 8, and 12 weeks. **(C)** The quantitative results of the new bone regrowth area in the defects of rats treated with electroacupuncture stimulation for 4, 8, and 12 weeks. The control group indicated the rats with the implanted GGT scaffold and without ES treatment. (The number of rats per group = 4, *p < 0.05.)

GGT scaffolds were transplanted into SD rats with skull bone defects (Supporting Figure S1). After 7 days, ES treatments were performed on the rats during 4, 8, and 12 weeks. To combine the effect of percutaneous ES with acupuncture, a stainless steel needle connected to the cathode was punctured at the acupoint Baihui (GV20), and another stainless steel needle connected to the anode was punctured at Fengfu (GV16) (Figure 2A). After performing ES for 4 weeks, (Figure 2B-i) shows that the gap between the implanted GGT scaffolds and native osseous tissues is still clearly observed by micro-CT for all groups. Furthermore, Von Kossa staining was used to evaluate the calcium deposits on the implanted GGT scaffolds in the paraffin tissue sections. Within 4 weeks, no significant calcium deposits were observed on the GGT scaffolds at the cathode (the cranial site) or the anode (the caudal site). On the contrary, numerous fibroblasts (blue arrows) were observed in the GGT scaffolds under all ES conditions. At 8 weeks, the 20 Hz/4 mA and 200 Hz/1 mA groups showed blurriness on micro-CT images between the GGT scaffolds and native osseous tissues from the cranial sites. This result indicates that new bone regeneration starts in the GGT scaffolds after 8 weeks of ES (Figure 2B-ii). Although the calcium deposit images show that the generation of fibroblasts is the major phenomenon, the significant calcium deposit behavior (red arrow) could be observed for the 20 Hz/4 mA and 200 Hz/1 mA groups, especially for the cranial sites in the 200 Hz/1 mA group. Next, (Figure 2B-iii) shows in comparison to the control group that the shape of the GGT scaffold is maintained only for the 2 Hz/1 mA group after ES treatment for 12 weeks. The scaffolds for the 20 Hz/4 mA, 200 Hz/1 mA, and 200 Hz/8 mA groups have an irregular shape and an unclear edge. Similar to the results in (Figure 2B-ii), the calcium deposits were observed for the 20Hz/4mA, 200Hz/1mA, and 200Hz/8 mA groups (red arrows), especially at the cranial sites in the 200Hz/1 mA group. Additionally, the degradation of GGT scaffolds (yellow arrows) also could be found at the cranial sites of the 200Hz/1 mA group. Moreover, through the quantitative results from micro-CT images, we observed that new bone regeneration occurred after ES treatment for the 20 Hz/4 mA, 200 Hz/1 mA, and 200 Hz/8 mA groups (Figure 2C). Moreover, compared with the control group, the rats treated with 200 Hz/1 mA ES had the fastest new bone regenerative behavior within 4 weeks and could achieve around 80% of the new bone regenerative area after 12 weeks.

### Mathematical Model for the Growth of Osteoclasts and Osteoblasts and the Remodeling of Bone Mass

**FIGURE 3:**
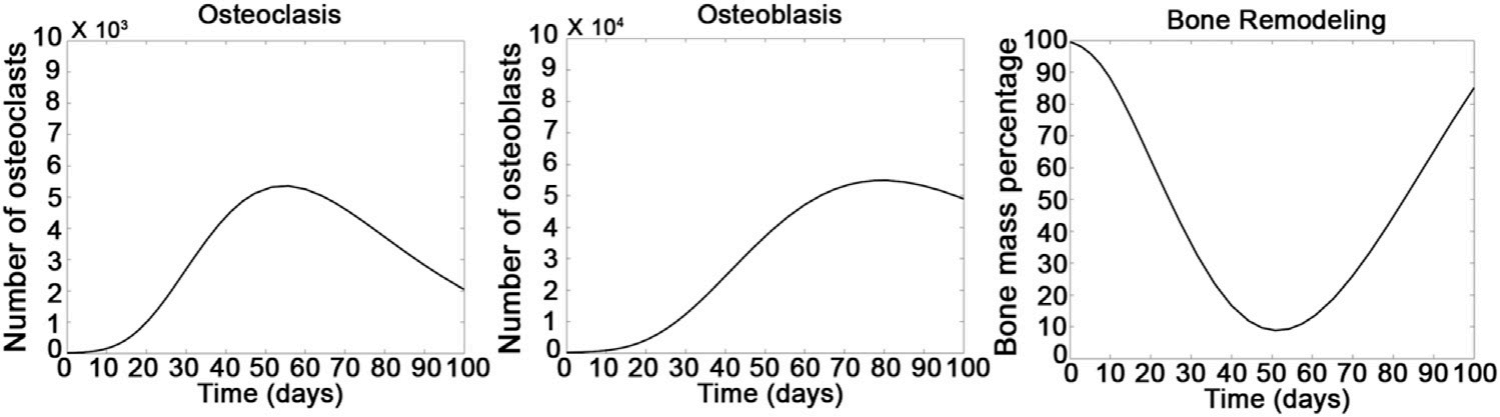
Computational calculation was designed for temporal patterning of the growth of osteoclasts, osteoblasts, and bone remodeling. Calculation was performed by setting the following parameters in Eq. (1–3). (*α*_1_ = 3, *α*_2_ = 4, *β*_1_ = 0.125, *β*_2_ = 0.035, g_11_ = 1.25, g_12_ = 0.75, g_21_ = -0.5, g_22_ = 0).

A mathematical model created by modifying the model from previous studies was used to predict the possible interactions between osteoclasts and osteoblasts in bone remodeling (Komarova et al., 2003; Ayati et al., 2010). First, we defined the beginning of treatment of the rats by ES as day zero. To fit the results of *in vivo* bone regeneration, we slightly modified the parameters from previous studies (Komarova et al., 2003; Ayati et al., 2010). The osteoclast (*a*_1_) and osteoblast (*a*_2_) production was set as three and four cells/day, and the removal of osteoclasts (*ß*_1_) and osteoblasts (*ß*_2_) was set as 0.125 and 0.035 cells/day, respectively. The g_11_ and g_21_ were set as 1.25 and 0.75, and the g_22_ and g_12_ were set as −0.5 and 0, respectively. Moreover, the activities of bone resorption (k_1_) and bone formation (k_2)_ were set as 0.001 and 0.000099%/(cell⋅day), respectively. As shown in Figure 3, the number of osteoclasts started to increase after 7 days of treatment and achieved a maximum number after 50 days of treatment; then, the number of osteoclasts decreased. Compared with the growth of osteoclasts, the number of osteoblasts increased when the number of osteoclasts slowly decreased back to a normal level, indicating that the growth of osteoblasts could be relative to the activity of osteoclasts after 8 weeks of ES treatment. Furthermore, consequent changes in bone regeneration showed a bone resorption behavior within the 8-weeks treatment and an increasing trend of bone formation after 8 weeks. These simulation results indicate that the resorption and regeneration of the bone are highly relative to the activities of osteoclasts and osteoblasts; and the computation model is in good agreement with the experimental results in Figure 2.

### ES Mechanism of Bone Regeneration

**FIGURE 4:**
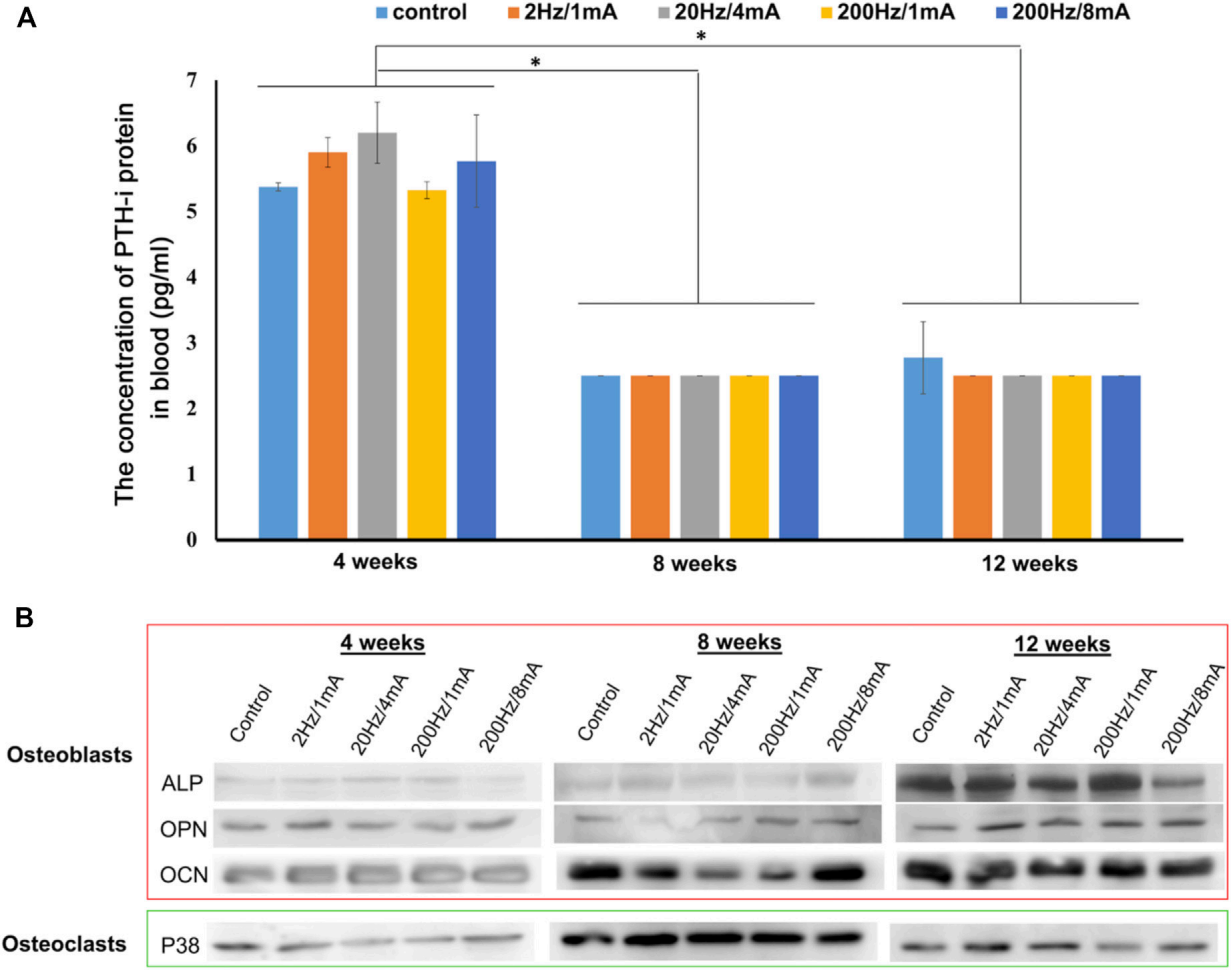
**(A)** Hematological analysis of the PTH-i expression of the bone-defected rats was treated with electroacupuncture for 4, 8, and 12 weeks. **(B)** The osteoclast activation protein (p38) and osteoblast differentiation/mineralization proteins (ALP, OPN, and OCN) in the bone-defected tissues after treating with electroacupuncture stimulation were analyzed by using the Western blot assay. The control group indicated the rats with the implanted GGT scaffold and without ES treatment. (The number of rats per group = 4, *p < 0.05.)

We measured the parathyroid hormone-intact (PTH-i) concentration in the blood from rats. As shown in Figure 4A, compared with the PTH released at 8 and 12 weeks, a higher level of PTH-i was observed only at 4 weeks for all groups, indicating that the bone defect at an early stage might induce the release of PTH-i. Subsequently, Figure 4B shows the expression by Western blot analysis of proteins related to bone regeneration such as osteocalcin (OCN), osteopontin (OPN), ALP, and p38. Compared with the expression at 4 weeks, the OCN protein level increased after 8 and 12 weeks of ES treatment. The OPN expression was significantly increased after 12 weeks of ES treatment. Noticeably, in comparison with the control group, the rats receiving ES treatment showed a higher OPN level. The results of the ALP expression in most groups show a similar trend to the OPN expression. Additionally, the expression of p38 protein could be found at the lowest level at 4 weeks. Then, the p38 expression reached the highest level after 8 weeks of ES treatment in all groups. Afterward, the p38 level started to decrease in all groups from 8 to 12 weeks. These results indicate that the expressions of bone regeneration-related proteins possess a time-dependent behavior after ES.

## Discussion

Remodeling in large bone defects is a long-term physiological process after bone injuries. Therefore, tissue engineering techniques provide the strategies to accelerate bone remodeling by using versatile chemical and physical treatments and has been of interest as new strategies for bone regeneration (Feng and McDonald, 2011). In the bone tissue engineering field, many biopolymers such as poly PCL and PLGA have been used as substitutes to promote the healing of bone fractures (Iaquinta et al., 2019). However, bone injuries in some cases are too complex so that the bone healing efficiency might be limited by using only one type of biomaterials. During the healing process of bone fracture, calcium ions play an indispensable role to enhance the cell–cell and cell–matrix interactions, regulating many cellular signaling pathways *via* SMAD and other routes (Lee et al., 2018). Therefore, calcium-based bioactive ceramics have been a popular chemical additive widely used to promote the osteogenic regeneration of the bone during the past decade (Nakamura et al., 2010). In addition to chemical additives, physical therapies such as mechanical-tension-stress or hyperthermia methods are also tested to accelerate the healing of the bone (Betts and Müller, 2014; Zhang et al., 2019). The physiological strain has been confirmed to induce the functionalization of stem or somatic cells (Ribas et al., 2017). Berman et al. reported in a short-term animal model of axial compressions that both structural and tissue level mechanical behaviors of the bone can be significantly increased through various loading (Berman et al., 2015), especially at a high loading. At the highest loading (12.4N), this strain level enhances the density of cortical and cancellous regions in the bone. Hyperthermia therapy is another physical method used to improve bone remodeling. Many substrates with light-to-heat conversion are able to absorb light in order to generate thermal energy (Gerbi et al., 2005; Serrat et al., 2015). For example, near-infrared (NIR) light irradiation-responsive black phosphorus (BP) nanosheets mixed with poly (lactic-co-glycolic acid) (BPs@PLGA) were used as an osteoimplant to treat bone defects in rats. Through the highly efficient NIR photothermal response of BPs, the BPs@PLGA significantly enhanced the expression of heat-shock proteins and promotes osteogenesis *in vitro* and *in vivo* (Tong et al., 2019).

In previous studies, we developed a TCP-crosslinked gelatin scaffold and a bone morphogenetic protein-2 (BMP-2)-immobilized gelatin/HA composite for promoting bone regeneration (Lin et al., 1998; Cheng et al., 2019). To investigate the effect of physical treatments, we also developed a method to remodel bone tissues *via* ES treatment, indicating that ES treatment stimulates bone tissue regrowth after 12 weeks (Yang et al., 2013). Acupuncture is another adjunctive physical therapy which has been developed during the several past decades. Recently, acupuncture in combination with electrical stimulation, called electroacupuncture, has been used in traditional Eastern medicine to promote disease recovery or to reduce the pain of patients (Lv et al., 2019). This observation inspired us to use an electroacupuncture method in combination with a GGT scaffold for repairing bone injuries. By combining advantages of Eastern and Western medicine, we hypothesized that electroacupuncture may provide with the GGT scaffold a dual significant effect (i.e., ES and acupuncture) to enhance the regeneration of the bone.

Osteoblast–osteoclast interactions are important to regulate new bone formation (Tanaka et al., 2005). During the bone remodeling process, osteoblasts and osteoclasts regulate each other’s maturation at various stages. For example, the communications between osteoblasts and osteoclasts promote the differentiation of pre-osteoclasts at an early stage during bone remodeling. The matured osteoclasts are responsible for resorbing the damaged and aged bone tissues at the injury site (Matsuo and Irie, 2008). Subsequently, osteoclasts further activate the osteoblasts to stimulate the new bone growth process (Wang et al., 2015). Noticeably, an imbalance between osteoclastic resorption and osteoblastic regrowth during bone remodeling may result in the occurrence of other bone diseases such as secondary osteoporosis (Chen et al., 2018). This information indicates that the expressions of osteoclastic resorption and osteoblastic regrowth contribute a key factor in the bone healing process.

To obtain the optimal frequency and current of ES treatment for bone regeneration in the animal model, we first set an *in vitro* OB/OC co-culture system to mimic the *in vivo* OB/OC environment. Then, through the connection of two OB/OC co-culture dishes by a salt bridge and electrodes, we recapitulate the current passing through tissues from the cathode to anode during ES treatment (Figure 1). According to a previous study (Tanaka et al., 2005), mature osteoclasts in the bone defect area participate in the resorption process at an early stage after injury. During the bone resorption process, osteoclasts release hydrogen ions to dissolve the minerals which may cause acidification of the microenvironment. Therefore, the negative ΔpH value reveals a low acidic environment in the OB/OC co-culture system without any ES (control group, Figure 1B). Interestingly, after treating the co-culture system with various frequency/ampere of ES, the pH value at the cathode site slightly increased following the increase of frequency/ampere, especially in the 200 Hz/8 mA group. As at the cathode a reduction reaction occurs and attracts the positive charge, the increase of frequency/current provides a more negatively charged environment which can neutralize the released positive hydrogen ions, causing a relative basic environment. Additionally, compared with the control group, we also found that not all cathode sites in the ES-treated culture groups could provide a relative alkaline environment, indicating that the frequency of the current higher than a critical point (around 2 Hz) might provide enough electrical ions to neutralize hydrogen ions. Moreover, Yuan, et al. and Zhang, et al. reported that an acidic pH environment reduces the proliferation, differentiation, and calcium absorption properties of osteoblasts, but it is able to enhance the activity of osteoclasts (Yuan et al., 2016; Zhang et al., 2017). The similar activation phenomena of osteoclasts and osteoblasts at the relative acidic and basic environments were also shown by the measurement of the osteoclastic TRAP and the osteogenic ALP levels (Figures 1C,D). Thus, it is reasonable to assume that the relative alkaline environment at the cathode site offers an appropriate condition for maturation of osteoblasts (Galow et al., 2017). According to the aforementioned information, the positive values of ΔpH and ΔALP were defined that the conditions may enhance the osteoblast activity, and the positive value of ΔTRAP was defined as the condition enhancing the osteoclast activity at the anode site in our study. Furthermore, we could classify the *in vitro* culture results from Figure 1 and Table 1 into three groups for different applications of the bone treated with ES: (1) “+++”/“+--” groups: the results may contribute to the inhibition of the osteoclast activity and slight enhancement of the osteoblast activity. (2) “+-+” groups: the results enable the resorption ability of osteoclasts at the initial stage of bone regeneration. (3) “++-”group: the groups observed for ES treatment with high frequency conditions which provide a desired effect on enhancing osteogenesis.

**TABLE 1:** The positive and negative expression of the *in vitro* OB/OC co-culture system after ES. n.s.: no statistically significant difference.

Groups	ΔpH value	ΔALP value	ΔTRAP value
Control	n.s.	+	+
1Hz/1 mA	n.s.	−	−
1Hz/4 mA	+	−	n.s.
1Hz/8 mA	+	−	+
2Hz/1 mA	+	+	+
2Hz/4 mA	−	−	−
2Hz/8 mA	−	−	−
20Hz/1 mA	+	−	−
20Hz/4 mA	+	+	−
20Hz/8 mA	+	−	+
200Hz/1 mA	+	+	n.s.
200Hz/4 mA	+	+	−
200Hz/8 mA	+	+	−

Therefore, we selected the groups from the *in vitro* results of the OB/OC co-culture system (Figure 1E), 2 Hz/1 mA, 20 Hz/4 mA, 200 Hz/1 mA, and 200 Hz/8 mA, which were the osteogenesis-enhancing conditions for stimulating bone regeneration in rats with bone defects. In the acupuncture method, acupoints Baihui (GV20) and Fengfu (GV16) have been demonstrated to induce the differentiation of cells and the healing of neural injuries (Nam et al., 2015; Salazar et al., 2017). However, there are few articles to discuss the relationship between these two acupoints and bone regeneration. Thus, the regeneration behavior of rats with the bone defect was further evaluated with an electroacupuncture technique.

Based on the animal model we set in the previous study (Yang et al., 2013), the stimulation of electroacupuncture is performed at the GV20 and GV16 acupoints *via* the 2 Hz/1 mA, 20 Hz/4 mA, 200 Hz/1 mA, and 200 Hz/8 mA parameters. Activation of osteoclasts at the early stage in the bone remodeling process is useful for removing and resorbing the damaged bone tissues (Shemesh et al., 2016). During the 12 weeks of ES treatment, there was no significant new bone regrowth process observed in all groups for the first 4 weeks (Figure 2B). Calcium ion signaling is an important factor in enhancing osteoclastic differentiation into active mature osteoclasts (Hwang and Putney 2011) because ES might directly activate the voltage-gated calcium channel in the cell membrane and then raise the level of intercellular calcium ions (Leppik et al., 2020). In this study, the implanted GGT scaffold contains TCP bioactive ceramics; it is reasonable to assume that the calcium ions on GGT scaffolds may activate the differentiation of osteoclasts. Thus, osteoclastic differentiation and resorption might be the only processes happening during the early stage of bone remodeling in our study (Figure 1C and Figure 2B).

Furthermore, the osteoclastic differentiation and resorption decrease are followed by the differentiation and maturation of osteoblasts starting in the next 4 weeks (Figure 2B). Compared to the group without ES, the cranial site in the bone defect received ES, providing a relatively high alkaline environment which can significantly promote osteogenesis for bone regeneration (Yuan et al., 2016; Zhang et al., 2017). In addition, our previous study had reported that the new bone regeneration area within 4 weeks was close to only 10% when the rats received the ES at 2 Hz without any acupuncture effect (Yang et al., 2013). Compared with our previous study (Yang et al., 2013), the rats treated with the ES at 2 Hz and acupuncture revealed a 20% new bone regeneration area within 4 weeks and around 40% new bone regeneration area after 12 weeks of ES treatment (Figure 2B), indicating a promoting effect of electroacupuncture on bone regeneration. Besides the acupuncture effect, the effects of the frequency/current level on *in vivo* bone regeneration were investigated by using the ES conditions. Similar to the new bone regrowth behaviors within 12 weeks (Figures 1B–D and Figure 2B), the higher frequency/current level enhances the new bone regrowth in the defect area after 12 weeks of ES treatment. Noticeably, the differentiation of osteoblasts and the bone regeneration area in the 200 Hz/1 mA group are higher than that in the 200 Hz/8 mA group (Figure 1E and Figure 2C). This can be attributed to the 200 Hz/8 mA treatment to excess electrical ions, leading to an alkaline environment which reduces the activation of osteoblasts (Figure 1B). In contrast, the ES condition at 200 Hz/1 mA may contribute to the adequate negative charge to the carboxylic group on gelatin, thereby enhancing the swelling of the GGT scaffold. Therefore, the cells can migrate into the swollen scaffold which might promote activation of osteoclasts *via* the TCP ceramide. Activated osteoclasts can then degrade the GGT scaffold, leading to a blurry edge of implanted scaffolds on the micro-CT images (Figure 2B). Therefore, these results indicated the combination of ES, acupuncture, and GGT enabling to enhancing new bone regeneration.

Next, we were eager to address the possible mechanism among the ES, acupuncture, and GGT in bone regeneration. Based on the results of new bone regeneration, a computation model was used to simulate and predict the bone regenerative behavior based on the regulation from OB/OC (Figure 3). g_11_ > 0 and g_21_ < 0 indicated positive feedback of osteoclast production in autocrine signaling and inhibition of osteoclast production in paracrine signaling that dominated a bone resorption process (Komarova et al., 2003; Ayati et al., 2010). The simulation model showed the osteoclast–osteoclast autocrine increased the osteoclast number at initial 4 weeks and achieved a maximum number at 8 weeks. Therefore, bone resorption was an initial process in the bone remodeling model. According to formula 1,2 in the mathematical model, the rate of bone formation was gradually increased because both the autocrine (g_22_) and paracrine (g_12_) of osteoblasts contributed positive feedback following the increase of remodeling time. Therefore, the number of osteoblasts gradually increased within 8 weeks and achieved maximum after the number of osteoclasts reached a normal level. Afterward, consequent changes in bone regeneration showed that the autocrine and paracrine among osteoclasts and osteoblasts regulated the bone resorption behavior within the 8-week treatment and an increasing trend of bone formation after 8 weeks. These simulation results indicated that the possible overall interactions between resorption and regeneration of the bone in our case and the computational model are in good agreement with the experimental results in Figure 2.

**FIGURE 5:**
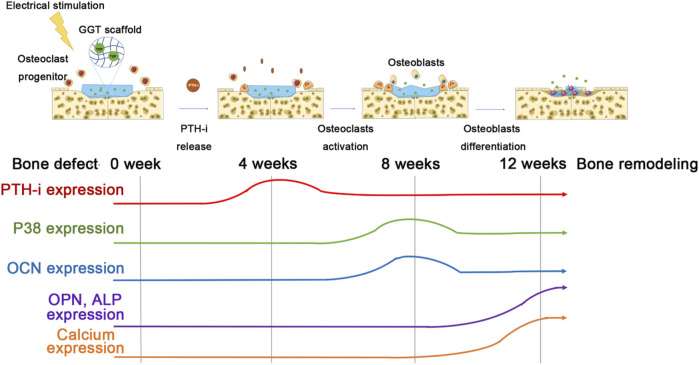
Schematic figure of the possible mechanism of the effect of electroacupuncture on the regulation of PTH-i, calcium, osteoclasts, and osteoblasts in the bone-defected rats.

Next, we further discussed the effect of the combination of ES, acupuncture, and GGT on bone remodeling in the molecular mechanism. Calcium plays a crucial role in bone regeneration (Aleem et al., 2016). When calcium concentration in the peripheral blood is low, PTH-i can be released from the parathyroid gland to promote calcium ion release from bones; then, the released calcium may further regulate the functions of osteoclasts and osteoblasts (Alkhiary et al., 2005). The acupuncture technique has been confirmed to accelerate the release of PTH-i from the parathyroid gland (Jincao et al., 2013). Therefore, the effect of electroacupuncture on the release of PTH-i leading to the activation of OB/OC and bone regeneration is a possible mechanism (Figure 5). During the first 4 weeks, electroacupuncture treatment might induce a neuronal activation (Nam et al., 2015; Salazar et al., 2017) and then promote the secretion of PTH-i from the parathyroid gland (Figure 4A). Compared with the control group, the treatment by electroacupuncture slightly increased the PTH-i concentration in blood. In general, electroacupuncture offers a temporal effect and requires regular repeats for sustained effects. Repetition of the electroacupuncture treatment resulted in a high PTH-i concentration in the blood for 4 weeks which may activate the osteoclasts and promote the osteoclast–osteoclast autocrine at the injured area. Upon activation, the osteoclasts express p38 from 4 to 8 weeks, indicating that osteoclasts may execute a bone resorption mechanism of debridement around the bone defect. Therefore, the possible PTH-i-p38 secreting mechanism regulated by electroacupuncture could dominate the increase of the osteoclast number and bone resorptions at the initial stages (Figures 2, 3).

After 8 weeks, although the PTH-i expression in the blood decreases, the p38 protein expression achieves a maximum level, indicating a strong activation of osteoclasts (Figures 3, 4). The increased expression of OCN after 8 weeks may be due to the fact that the activation of osteoblasts and the release of PTH-i provide a paracrine effect for the activation of osteoblasts (Tanaka et al., 2005). Also, compared with other ES groups, the OPN expressions in the 20 Hz/4 mA and 200 Hz/1 mA groups are significantly higher than others. This observation suggests that the new bone regeneration process has been started between 4 and 8 weeks (Figure 3), especially in the 200Hz/1 mA group (Figure 2C). Next, following the 12-week electroacupuncture treatment, the end of the osteoclastic activity may cause the reduction of the p38 level. Similarly, the expression of PTH-i also showed a stable low level, indicating that the effect of electroacupuncture on the PTH-i expression level may be related to the activation of osteoclasts at the early stage of bone remodeling. Then, the combination of a low PTH-i expression level and degradation of the GGT scaffold maintains the essential calcium concentration in the blood for bone remodeling [Figure 2B-iii] and (Supporting Figure S2), thereby promoting the maturation and mineralization of osteoblasts and new bone regeneration (Figure 2, Figure 3, and Figure 4B).

## Conclusion

In this study, we successfully provide a concept to combine a GGT scaffold and an electroacupuncture technique for new bone regeneration. The stimulation by electroacupuncture provides a potential strategy to raise the PTH-i level in blood to activate osteoclasts at the early stage of bone remodeling. Furthermore, the activation of osteoclasts not only executes a debridement process but also promotes the maturation and mineralization of osteoblasts for new bone regeneration at the middle and late stages. This work shows the feasibility of combining traditional Eastern and Western medicine to stimulate cell interactions, organizations, possible functions, and bone remodeling in bone tissue engineering.

## Data Availability

The original contributions presented in the study are included in the article/Supplementary Material; further inquiries can be directed to the corresponding author.
