# Supplementary material for: Remodeling Effects of the Combination of GGT Scaffolds, Percutaneous Electrical Stimulation, and Acupuncture on Large Bone Defects in Rats
Source: Front Bioeng Biotechnol. 2022 Feb 28;10:832808. doi: 10.3389/fbioe.2022.832808 (PMC8919371; doi:10.3389/fbioe.2022.832808)
Supplement: Supplementary file 1 [file DataSheet1.docx]

Supporting Figures:


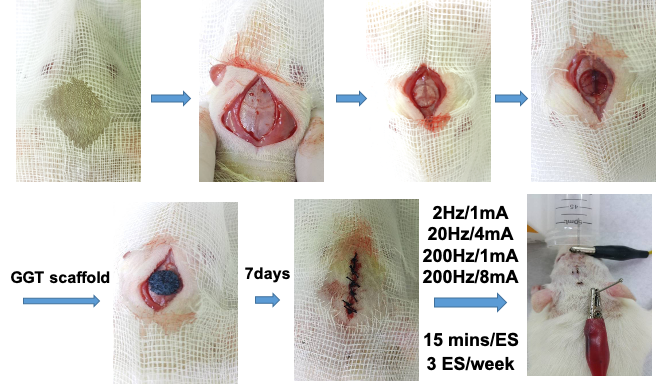


Supporting Figure S1. The procedure of the bone-defected rats was implanted GGT scaffolds and treated with electroacupuncture stimulation.


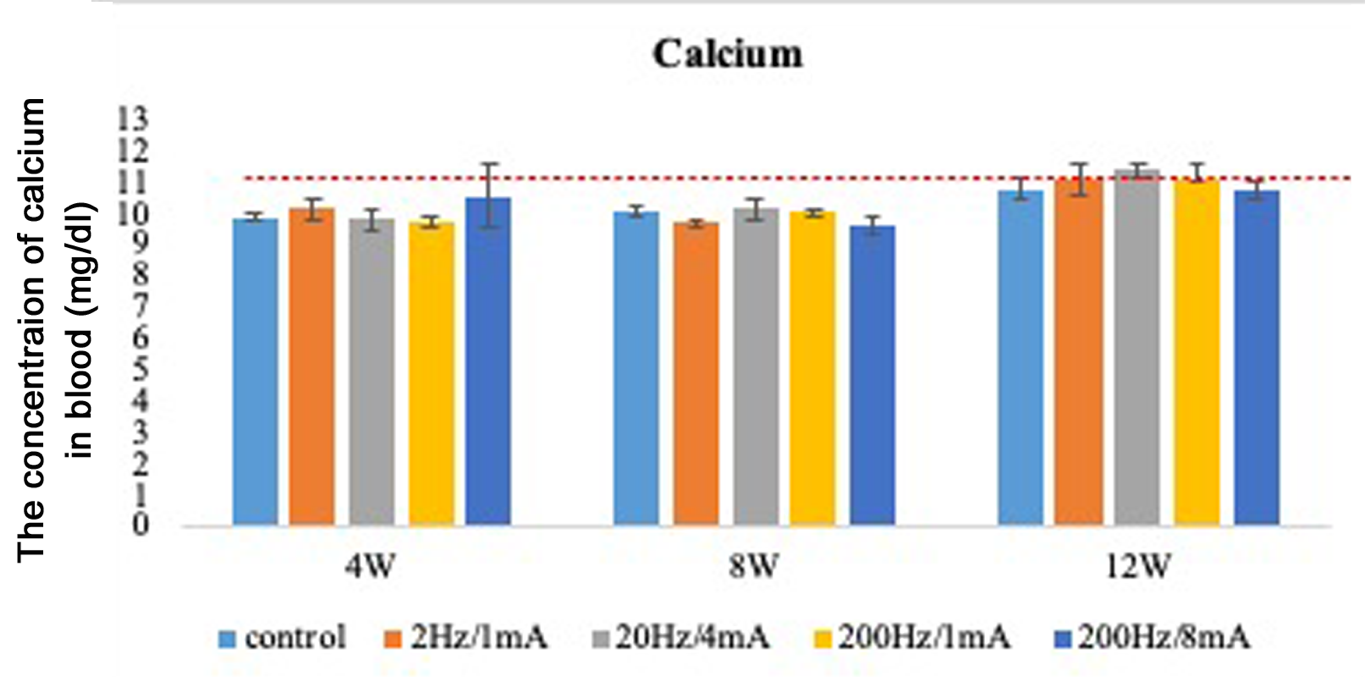


Supporting Figure S2. The calcium expression in blood of the bone-defected rats were measured by using hematological analysis after 4, 8 , and 12 weeks.
